# The acute and prolonged effects of 20-s static stretching on muscle strength and shear elastic modulus

**DOI:** 10.1371/journal.pone.0228583

**Published:** 2020-02-06

**Authors:** Shigeru Sato, Ryosuke Kiyono, Nobushige Takahashi, Tomoichi Yoshida, Kosuke Takeuchi, Masatoshi Nakamura

**Affiliations:** 1 Department of Physical Therapy, Niigata University of Health and Welfare, Niigata, Japan; 2 Department of Physical Therapy, Faculty of Rehabilitation, Kobe International University, Hyogo, Japan; 3 Institute for Human Movement and Medical Sciences, Niigata University of Health and Welfare, Niigata, Japan; University of L'Aquila, ITALY

## Abstract

**Introduction:**

Static stretching (SS) is commonly performed as part of warm-up routine. However, only few previous studies have reported on the effects of short-duration SS, which is often used in the sports field. The purpose of this study was to investigate the acute and prolonged effects of 20-s SS on isokinetic contraction muscle strength, range of motion (ROM), and the shear elastic modulus.

**Method:**

Twenty male volunteers participated in this study. The ROM and both concentric and eccentric contraction muscle strengths were measured using a dynamometer. In addition, the shear elastic modulus of medial gastrocnemius muscle in dominant leg was measured by ultrasonic shear wave elastography. The participants visited the laboratory on four occasions each separated by >3 days. The first visit was a familiarization trial, and the subsequent three visits included the following experimental conditions in a random order. All measurements were performed prior to and immediately after SS or 5 min and 10 min after 20-s SS.

**Results:**

The results of this study showed that the ROM was significantly increased SS intervention in all conditions compared with prior to SS intervention. In addition, ROM was significantly higher post SS and 5 min after SS than 10 min after SS. However, there were no significant interaction effects for isokinetic contraction muscle strength and the shear elastic modulus

**Conclusion:**

In the sports field, from the point of performance, a 20-s SS intervention could be a useful technique before exercise because it increases ROM and does not decrease maximum torque.

## Introduction

In athletic and rehabilitation settings, static stretching (SS) is commonly performed as part of the warm-up routine to increase joint flexibility, improve performance, and reduce injury risk. However, in recent years, many studies have reported negative effects of SS on athletic performance, such as the reduction of maximum muscle strength and power [1–4]. In addition, review articles have reported on the negative effect of 45- to 90-s SS in one muscle group prior to exercise such as decreased maximum muscle strength and performance [1–4]. Therefore, considering the actual exercise duration on sports field, Behm and Chaouachi (2011) recommended that SS duration before exercise should be <30 s. In addition, the previous study reported that the percentage of performing <20 s SS in one muscle group was 73% in sports field, which was larger than that of >20 s SS duration [5]. In addition, stretching duration of <20s has already been reported in previous surveys on strength and conditioning coaches of the National Hockey League, Major League Baseball, and National Football League [6–8]. Therefore based on previous reviews [1–4], short-duration SS (<20 s) may not necessarily lead to a reduction in the maximum strength and performance on sports field. However, only a few studies [9, 10] have reported the effects of short-duration SS (<20 s) on muscle strength and performance in the sports field. Therefore, further research is required to investigate the effect of short-duration SS on exercise performance.

Adverse effects of SS on maximum muscle strength vary according to the type of muscle contraction induced. Behm et al. have previously reported that the reduction rate of isometric contraction muscle strength is larger than that of isokinetic contraction (i.e., concentric and eccentric contractions) muscle strength, which is modulated by SS duration [2]. Additionally, Kay and Blazevich reported that the isokinetic concentric contraction muscle strength of the plantar flexor muscle group decreased after 3-min SS [11]. In addition, Cramer et al. investigated the effect of 2-min SS on the isokinetic eccentric contraction muscle strength of the knee extension muscle group and found no significant change in the eccentric contraction muscle strength after 2-min SS [12]. Although the effect of long-duration SS (2–3 min) on concentric and eccentric contraction muscle strengths has previously been investigated, there are no reports investigating the effect of <20-s SS on the isokinetic contraction muscle strength. As <20-s SS is often used on sports field, studies evaluating the effect of short-duration SS on the concentric and eccentric contraction muscle strengths are required.

In addition to the acute effect of SS duration on maximum strength and performance, the prolonged effect of SS is also vital to athletes and coaches on sports field. Ryan et al. investigated the effect of 2, 4, and 8-min SS on the plantar flexor group and reported that the decrease in muscle strength and increase in ROM recovered within 10 min [13], and the decrease in musculotendinous stiffness recovered within 10 min after 2-min SS or 20 min after 4- or 8-min SS [14]. In addition, Mizuno et al. investigated the effect of 5-min SS on the plantar flexor group and reported that the decrease in muscle strength recovered within 10 min [15], but the increase in range of motion (ROM) was sustained for >30 min after SS [16]. However, previous studies investigated the prolonged effect of SS, with <2 min for one muscle group. As described above, because <20 s of SS is often used on sports field, the prolonged as well as acute effect of short-duration SS is also investigated.

The shear elastic modulus, which is used to quantitatively evaluate muscle stiffness *in vivo*, was measured using shear wave elastography (SWE). The acute effect of SS on the shear elastic modulus was reported in previous studies [17–21]. Specifically, Akagi et al. investigated the effect of SS on dorsiflexion (DF) ROM, passive stiffness, the shear elastic modulus of the medial head of gastrocnemius muscle (MG), and the lateral head of gastrocnemius muscle. Reportedly, SS causes increased ROM, decreased passive stiffness, and shear elastic moduli of both MG and lateral head of gastrocnemius muscle [17]. Moreover, according to Nakamura et al., the shear elastic modulus of MG was decreased after 2-min SS [18]. However, as no previous studies have investigated the effect of ≤20-s SS on the shear elastic modulus for MG and the consequent prolonged effect of SS on shear elastic modulus, such studies are warranted.

As mentioned, short-duration SS before exercise is assumed to be a useful technique for reducing the maximum muscle strength, which recovers after a few minutes of SS along with an increase in ROM and a subsequent decrease in the shear elastic modulus by short-duration SS often used on sports field. Therefore, we aimed to investigate acute and prolonged effects of 20-s SS on the isokinetic contraction muscle strength, ROM, and shear elastic modulus. It has been considered that there is a dose-response relationship between SS duration and the effect of SS. We hypothesized that there were no significant changes in muscle strength and shear elastic modulus after short duration (20-s) SS, whereas ROM was increased significantly.

## Methods

### Experimental design

**Fig 1:**
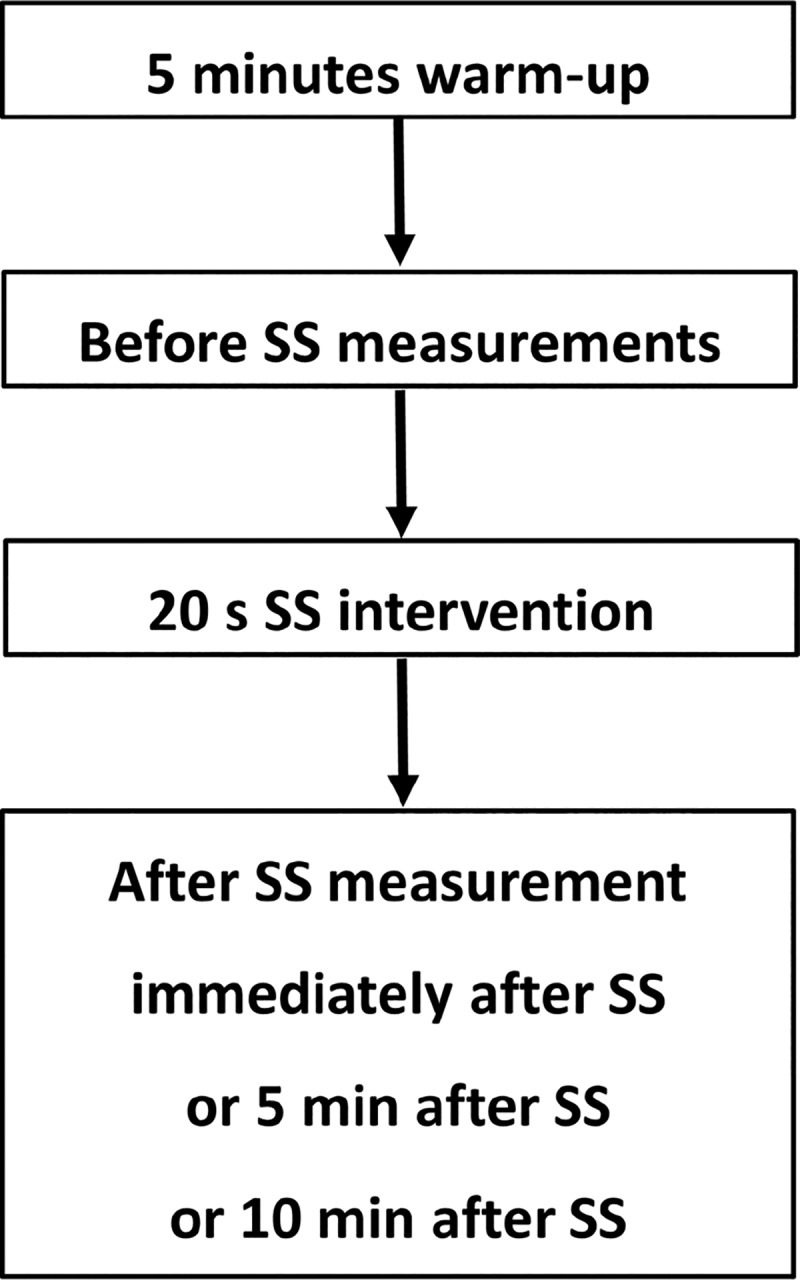
Experimental design flowchart. The after SS measurement in three conditions (immediately after or 5 min after or 10 min after SS) were performed at random order with an interval of >3 days. SS: static stretching.

We used a randomized repeated-measures experimental design to investigate the effects of SS on the isokinetic contraction muscle strength, ROM, and shear elastic modulus of MG in the dominant leg. Previous studies measured the dominant leg [16, 22]; hence, it was also measured in this study. The dominant leg was defined as the preferred leg for kicking a ball. Participants visited the laboratory on four occasions with an interval of >3 days. The first visit was a familiarization trial, and on the subsequent three visits, the participants were subjected to the following experimental conditions in random order (immediately after or 5 min after or 10 min after SS, Fig 1). Before experimental trials, all participants practiced measuring the isokinetic contraction muscle strength and ROM to ensure comfort and familiarity of the procedures and to minimize any potential learning effects.

Based on a previous study [23], participants performed a 5-min warm-up on an exercise cycle at 60 rpm with 50 W in all conditions. Then, we measured DF ROM, the ankle isokinetic contraction muscle strength (i.e., concentric and eccentric contraction torques), and the shear elastic modulus before and after SS for plantar flexors. In accordance with a previous study [24], we performed all measurements prior to (pre) and immediately after SS, or 5 and 10 min after SS. Participants remained in a resting sitting position on the isokinetic dynamometer (Biodex System 4.0; Biodex Medical Systems, NY, USA) for 5 and 10 min after SS.

### Participants

The participants were 20 healthy, nonathletic, male volunteers (age, 21.0 ± 0.2 years; height, 169.4 ± 4.8cm; body mass, 62.8 ± 4.1 kg). We excluded those with a history of neuromuscular disease or lower extremity musculoskeletal injury. During the experimental period, we instructed all participants to avoid strength training and stretching of the lower limbs. We provided all participants detailed information of the procedures and purpose of the study, following which the patients provided written informed consents. The Ethics Committee of the Niigata University of Health and Welfare, Niigata, Japan (Procedure #17677) approved the study and complied with the requirements of the Declaration of Helsinki.

### Assessment of DF ROM

We securely seated the subjects in the isokinetic dynamometer chair (Biodex System 4.0) at 0° knee angle (i.e., the anatomical position), with adjustable belts fixed over the trunk and pelvis. We reclined them (55° hip angle; 0° full extension) to prevent tension at the back of the knee. Then, the dynamometer footplate was manually moved by an examiner, starting from an ankle angle at 30° plantarflexion to a dorsiflexion angle just before the subjects started to feel discomfort or pain [17]. The angle just before this point was defined as the DF ROM angle. All measurements were repeated twice, and the average value was used for analysis.

### Assessment of the shear elastic modulus of MG

In this study, we measured the shear elastic modulus of MG using ultrasonic SWE (Aplio 500, Toshiba Medical Systems, Tochigi, Japan) using a 5–14 MHz linear probe at an angular position of 10° dorsiflexion and using positions similar to those used during DF ROM measurement. Then, we measured the shear elastic modulus of MG at 30% of the lower leg length from the popliteal crease to the lateral malleolus near the point of the maximal cross-sectional area of the lower leg [17, 18]. We obtained elastgraphic images in duplicate in the state of the long-axis image of MG and obtained ultrasound images using a custom, image analysis software (MSI Analyzer version 5.0; Rehabilitation Science Research Institute, Japan). We drew the quadrangular the region of interest (ROI) as large as possible within the color-coded area of the elastographic images while accounting for the artifact from aponeurosis. The software automatically calculated the average value of Young’s modulus in the quadrangular ROI. Based on previous studies [19, 20], we calculated the shear elastic modulus by dividing the obtained Young’s modulus by 3. We obtained the elastographic image of MG twice, and the average value of shear elastic modulus obtained from these images was used for analysis [17, 18, 25].

### Assessment of isokinetic muscle strength

We measured the isokinetic muscle strength of plantarflexor using the dynamometer (Biodex System 4.0). The measurement position was similar to that used for assessing DF ROM and the shear elastic modulus. The motion range was from 20° of plantar flexion to 10° of dorsiflexion, with an angular velocity of 30°/s [26]. In addition, we applied concentric and eccentric contraction protocols five times in each sequence. Throughout the measurement, we strongly, verbally encouraged the participants during contraction to promote maximal effort. We measured the maximum torque during both concentric and eccentric contractions. The concentric and eccentric strengths are assumed to reflect muscle power rather than the isometric strength; therefore, the effect of short-term SS on concentric and eccentric strengths is investigated in this study.

### SS intervention

We applied the SS protocol using a dynamometer in the sitting position with the knee extended, similar to position used during DF ROM and shear elastic modulus measurements. The examiner manually moved the footplate of the dynamometer (Biodex System 4.0), starting from 0° plantar flexion to DF ROM. The examiner held the ankle joint at DF ROM for 20 s. In all conditions, only one set of SS was performed for 20s, and the examiner instructed the participants to rest.

### Reliability of measurements

All variables were measured twice, before and after 20s of sitting position and compared with measurements obtained immediately after an SS to assess the test–retest reliability. Out of 20, 7 participants were randomly recruited to determine the reliability of measurements (age, 21.0 ± 0.0 years; height, 168.7 ± 5.7 cm; body mass index, 62.6 ± 3.9 kg).

### Statistical analysis

We used SPSS (version 24.0; SPSS Japan Inc., Tokyo, Japan) for statistical analysis. Measurement reliability was assessed using the intraclass correlation coefficient [1,1]. Based on the reliability coefficients, the standard error of measurement (SEM) was calculated (SEM = SD √1 − ICC) for each measurement [27, 28]. For all variables, we performed a two-way repeated measure analysis of variance (ANOVA) (test time [before vs. after SS] and condition [after SS vs. 5 min vs. 10 min condition]) to analyze the interaction and main effect. Furthermore, as a post hoc test, we determined significant differences between before and after SS measurements using a paired *t*-test in each condition. Additionally, we used the Bonferroni multiple comparison test to determine significant differences among the conditions in both before and post SS. If there were significant changes, effects sizes (ES) were calculated as the differences between before and post SS mean divided by before stretching standard deviation [29]. In addition, an ES of 0.00–0.19 was considered as trivial, 0.20–0.49 as small, 0.50–0.79 as moderate, and ≥ 0.80 as large [29]. We assumed differences to be statistically significant at an alpha level of p < 0.05. We have indicated descriptive data as means ± standard deviation.

## Results

Measurements of reliability assessments are summarized in Table 1 and confirmed in this study. Table 2 presents all variables in all conditions. The repeated two-way ANOVA indicated a significant interaction effect for DF ROM (p = 0.004, F = 6.517, η_p_^2^ = 0.255) and a significant main effect of time (P < 0.01, F = 64.32, η_p_^2^ = 0.772). However, there was no significant main effect of condition (P = 0.068, F = 2.89, η_p_^2^ = 0.132). The post hoc test revealed a significant increase in DF ROM after SS in all conditions compared with that before SS (ES = 0.93, 0.49, 0.24, respectively). In addition, post hoc test revealed that there were no significant difference in DF ROM before SS among all conditions, whereas DF ROM was significantly higher immediately after SS compared with 10 min after SS. We observed no significant interaction effects for the isokinetic contraction muscle strength and shear elastic modulus (concentric contraction: p = 0.373, F = 1.013, η_p_^2^ = 0.051; eccentric contraction: p = 0.917, F = 0.087, η_p_^2^ = 0.005; shear elastic modulus: p = 0.785, F = 0.243, η_p_^2^ = 0.013). In addition, no significant main effect was observed in time (concentric contraction: p = 0.796, F = 0.069, η_p_^2^ = 0.004; eccentric contraction: p = 0.303, F = 1.119, η_p_^2^ = 0.056; shear elastic modulus: p = 0.256, F = 1.37, η_p_^2^ = 0.067) and condition (concentric contraction: p = 0.634, F = 0.461, η_p_^2^ = 0.024; eccentric contraction: p = 0.932, F = 0.07, η_p_^2^ = 0.004; shear elastic modulus: p = 0.785, F = 0.243, η_p_^2^ = 0.013).

**Table 1:** Reliability assessment of DF ROM and muscle stiffness measurements.

	Test	Retest	ICC (1,1)	SEM
DF ROM (°)	21.9 ± 5.8	23.8 ± 9.1	0.845 P < 0.01	2.9
Shear elastic modulus (kPa)	26.9 ± 18.9	24.0 ± 19.7	0.952 P < 0.01	4.2
Concentric contraction torque (Nm)	137 ± 16	137 ± 20	0.865 P < 0.01	6.6
Eccentric contraction torque (Nm)	215 ± 25	215 ± 26	0.955 P < 0.01	5.4

Data presented as mean ± standard deviation.

DF, dorsiflexion; ROM, range of motion; ICC, intraclass correlation coefficient; SEM, standard error of measurement

**Table 2:** Variables before and after static stretching (SS) in all conditions.

	Immediately after SS		5 min after SS		10 min after SS	
	before	after	before	5 min	before	10 min
DF ROM (°)	23.7 ± 5.8	29.4 ± 7.4*	23.1 ± 6.5	26.1 ± 7.2*	23.3 ± 6.1	24.8 ± 5.3*^†^
Shear elastic modulus (kPa)	19.5 ± 14	17.3 ± 9.7	18.0 ± 7.6	20.1 ± 9.6	18.0 ± 16	18.0 ± 13
Concentric contraction torque (Nm)	136 ± 20	138 ± 18	137 ± 20	138 ± 19	140 ± 20	139 ± 20
Eccentric contraction torque (Nm)	222 ± 27	223 ± 27	221 ± 32	224 ± 31	220 ± 30	222 ± 39

*: p < 0.05: significant difference between before and after, 5 min after, and 10 min after SS

†: p < 0.05: significant difference between immediately after and 10 min after SS

DF ROM: dorsiflexion range of motion

## Discussion

We investigated the acute and prolonged effects of 20-s SS on DF ROM, the isokinetic ankle joint plantar flexor torque, and the shear elastic modulus of MG. The two major findings of this study are the following: 1) 20-s SS increased ROM, and this increase was sustained for 10 min after SS and 2) the isokinetic muscle strength and shear elastic modulus showed no significant differences after 20-s SS. Although many studies have investigated the acute and prolonged effect of long-duration SS on the muscle strength and shear elastic modulus [15, 17, 18, 20, 21, 24], to the best of our knowledge, our study is the first to investigate the acute and prolonged effect of 20-s SS on the isokinetic contraction muscle strength and shear elastic modulus. These findings have implications on pre-exercise stretching on sports field.

Results of two-way ANOVA demonstrated a significant interaction for DF ROM and time as well as a significant main effect for time. Based on the results of the post hoc test, DF ROM significantly increased after SS compared with that before SS in all conditions. In addition, DF ROM was significantly higher immediately after SS and 5 min after SS than that 10 min after SS. This result is consistent with that of previous studies [13, 16], which reported that 20-s SS was effective in promptly increasing DF ROM. In addition, our results suggested that the increase in DF ROM by 20-s SS was reduced by 5–10 min after SS. Additionally, they investigated the effect of 5-min SS on the plantar flexor muscle group and reported that the increase in DF ROM was sustained for >30 min after SS [16]. Similarly, Hatano et al. investigated the prolonged effect of 5-min SS on hamstrings and reported that the increase in knee extension ROM was sustained for >30 min after SS [24]. However, in the current study, the increase in DF ROM decreased by up to 10 min, but DF ROM was significantly different at 10 min after SS compared with that at immediately or 5 min after SS. Thus, the sustained effect was smaller on sports field than that in previous studies [16, 24]. There was a difference between the two studies with respect to SS duration: 5 min in previous studies compared to 20 s in this study. As mentioned above, 20-s SS is effective in promptly increasing DF ROM, but the effect is diminished within 5–10 min.

There was no significant interaction and main effect of the shear elastic modulus, suggesting that 20-s SS has no effect on the shear elastic modulus. Previous studies have demonstrated a significant decrease in the shear elastic modulus of MG after 2- and 6-min SS [17, 18, 20, 21]. Conversely, this study reported no significant change because SS duration (20 s) used in the current study was much shorter than that used in previous studies [17, 18, 20, 21]. Therefore, we consider that 20-s SS is insufficient to decrease the shear elastic modulus in the medial GM. The previous study identified that increased muscle stiffness may increase the risk of muscle strain and damage [30]. Therefore, in the context of preventing muscle strain and damage, SS duration of 20 s is insufficient.

In addition, we observed no significant interaction or main effect, suggesting that 20-s SS does not affect the concentric or eccentric contraction torque. To explain stretching-induced force deficit, a previous study proposed two primary factors: neural factors (altered motor control strategies or reflex sensitivity) and mechanical factors (changes in muscle stiffness) [12]. Kay et al. reported that 3-min SS resulted in a change in the neurological factors and a decrease in the concentric contraction torques [11]. In addition, mechanical changes of the muscle are involved in stretching-induced force deficit after SS because the muscle activity did not decrease after 60-s SS [31]. In the current study, we did not observe a decrease in the concentric contraction torque after SS. This is likely due to a lack of neurological and mechanical changes in the relatively short SS duration of 20-s, as well as an unaffected afferent contraction strength. Conversely, a previous study suggested that the eccentric contraction torque was not related to the stiffness of the muscle tendon complex [32]. According to Cramer et al., the stiffness of the muscle tendon complex decreased after 2-min SS, without a concurrent decrease in the eccentric contraction torque [12]. In our study, the eccentric contraction torque did not decrease after 20-s SS, consistent with the previous study [12]. Therefore, the adverse effect of SS on eccentric contraction was minimal regardless of SS duration. However, since there are no previous studies comparing the difference of SS duration on the eccentric contraction torque, future studies are needed to investigate the effects of different SS durations on the eccentric contraction muscle strength.

This study has few notable limitations. First, we did not include a control condition without stretching intervention. Second, we only examined the shear elastic modulus of MG. Similar acute effects of SS might not be observed in other muscles. Third, we only assessed isokinetic contractions and not functional athletic performance, such as jump height. Further studies are required to investigate the effect of 20-s SS in other muscles and on athletic performance.

## Conclusions

We investigated the acute and prolonged effect of 20-s SS on ankle joint DF ROM, the shear elastic modulus, and the isokinetic ankle joint plantar flexor torque. This study suggests that 20-s SS is effective in immediately increasing DF ROM. The increase in DF ROM significantly diminishes at 10 min after SS. In addition, we found that 20-s SS did not affect the shear elastic modulus and concentric or eccentric contraction torque. When applied in the context of on-field performance, a 20-s SS intervention could be useful to increase ROM without reducing the maximum torque.

## Supporting information

S1 File Data set in all participants. (XLSX)
